# Regional atrophy, cellular plasticity, and regenerative potential in irradiated murine salivary glands

**DOI:** 10.2340/1651-226X.2025.44012

**Published:** 2025-07-23

**Authors:** Inga Solgård Juvkam, Olga Zlygosteva, Olaf Joseph Franciscus Schreurs, Nina Jeppesen Edin, Hilde Kanli Galtung, Eirik Malinen, Tine Merete Søland

**Affiliations:** a Department of Radiation Biology, Institute for Cancer Research, Oslo University Hospital, Oslo, Norway; b Institute of Oral Biology, Faculty of Dentistry, University of Oslo, Oslo, Norway; c Department of Physics, Faculty of Mathematics and Natural Sciences, University of Oslo, Oslo, Norway

**Keywords:** Radiotherapy, fractionation, late effects, salivary glands, mice, histology, cellular plasticity

## Abstract

**Background and purpose:**

Radiotherapy of head and neck cancer may cause detrimental late side effects such as fibrosis and hyposalivation. We investigated mouse salivary glands after fractionated irradiation, with the aim to elucidate cellular plasticity and potential regeneration.

**Methods:**

12-week-old female C57BL/6JRj mice were irradiated with X-rays to a total dose of 66 Gy, given in 10 fractions over 5 days. The radiation field covered the oral cavity and major salivary glands. The submandibular (SMG), sublingual (SLG), and parotid glands (PG) were dissected at day 100 after finishing irradiation. Using different histological staining techniques, morphological, cellular, and molecular changes were investigated in irradiated and control SMG, SLG, and PG.

**Results:**

Atrophy of acinar cells was observed in irradiated SMG and SLG, but not in PG. Surprisingly, the acinar atrophy was confined to one region of each irradiated gland. These atrophic regions showed distinct cellular and molecular compositions compared to non-atrophic regions and control glands. Increased expression of the ductal cell markers keratin 5 and 19 (K5 and K19), along with increased percentages of proliferating myofibroblasts, fibroblasts, leukocytes, and acinar cells (Nkcc1^+^) were observed in the atrophic regions compared to controls. In addition, some of the K19^+^ and K5^+^ duct-like cells also co-expressed Nkcc1.

**Interpretation:**

Through a detailed histological assessment of the cellular and molecular changes in the major salivary glands of irradiated mice, we observed signs of cellular plasticity where ductal cells adopt an acinar cell phenotype upon irradiation. This suggests a regenerative potential of salivary glands after irradiation.

## Introduction

Head and neck cancer patients are often treated with radiotherapy, which may cause severe late side effects such as salivary gland fibrosis and hyposalivation due to radiation-induced damages to the salivary glands. Hyposalivation has detrimental effects on oral health causing loss of taste, difficulties in eating, speaking, and swallowing, as well as increased incidence of caries and fungal infections, all with negative consequences for the patients’ quality of life [1, 2].

The submandibular (SMG), sublingual (SLG), and parotid (PG) glands constitute the three pairs of major salivary glands in both humans and rodents. The salivary glands contain saliva-producing mucous and serous acinar cells, different (intercalated, striated, and excretory) ductal cells, myoepithelial cells, blood vessels and nerves that all can be affected by radiation [1, 3]. In contrast to the long-standing assumption that a stem cell population supports salivary gland homeostasis, emerging evidence indicate that the acinar and ductal cell populations are maintained by lineage-restricted progenitor cells [4–6]. After stress or injury, however, cellular plasticity has been observed between the different cell compartments in the salivary glands, where ductal cells adopt an acinar cell phenotype possibly to aid in regeneration of the damaged acinar cell population [5, 7–11]. However, the knowledge regarding cellular plasticity in salivary glands following radiation injury is still limited and needs to be further elucidated.

We have previously shown that irradiation of the head and neck of C57BL/6JRj mice resulted in increased salivary gland fibrosis and reduced saliva production [12, 13]. In the present study, our aim was to examine the mouse salivary glands 100 days after irradiation, through different histological and immunofluorescent staining techniques. We aimed at investigating radiation-induced morphological, cellular, and molecular changes to further understand cellular plasticity and potential regeneration in the salivary glands after radiation damage.

## Material and methods

### Animals

Nine-week-old C57BL/6JRj female mice were purchased from Janvier (Janvier Labs, France), kept in a 12-h light/12-h dark cycle under pathogen-free conditions and fed a standard commercial fodder with water given *ad libitum*. Standard housing with nesting material and refuge was provided. All experiments were approved by the Norwegian Food Safety Authority (ID 27931) and performed in accordance with directive 2010/63/EU on the protection of animals used for scientific purposes. At the onset of irradiation, animals were 12 weeks old.

### Irradiation procedure

Using the preclinical model recently published [14], mice were irradiated with 10 fractions over 5 days (8 am and 4 pm) with a Faxitron Multirad225 irradiation system (Faxitron Bioptics, Tucson, AZ, USA) using following X-ray settings: 100 kV X-ray potential, 15 mA current and 2 mm Al filter with a dose rate of 0.75 Gy/min. Mice were randomly assigned to either sham treatment or 10 × 6.6 Gy (*n* = 10 for each treatment group). The dose was chosen based on previous studies using different doses, where the chosen dose was found to be the optimal dose to observe both acute and late normal tissue damage [13, 14]. The radiation field was designed to include the oral cavity, pharynx, and major salivary glands while avoiding exposure to the eyes and brain. A custom-made lead collimator and the same person positioning each mouse was used to ensure proper irradiation of the same radiation field for all mice and fractions (Supplementary Figure S1). Mice were under gas anaesthesia (4% Sevoflurane + O_2_) during the irradiation procedure.

### Experimental protocol

On days 0–4, fractionated irradiation was given twice a day, as explained above. The maximum follow-up period in this study was 100 days after finishing irradiation, in order to ensure full documentation of late tissue responses in the salivary glands, as fibrosis has previously been observed in mice at day 90 after single dose irradiation [15, 16]. At day 100 after irradiation, euthanasia was performed through overdose of an anaesthetic (Pentobarbitol, Exagon® Vet) by intraperitoneal injection. The SMG, SLG, and PG were collected from each animal. All tissues were fixed in 10% formalin for 24 h before undergoing dehydration and embedding in paraffin.

### Staining of tissues

For hematoxylin and eosin (HE), Alcian blue (AB), Masson trichrome (MT), and immunofluorescence (IF) staining, tissue sections of 4–6 µm, were cut (Leica RM2155 microtome) and placed on microscopy glass slides (Superfrost Plus, Thermo Fisher) before deparaffination and hydration (for HE, AB, and MT staining, see Supplementary materials). After staining, HE, AB, and MT sections were dehydrated and mounted with xylene-based mounting medium (Pertex, Chemi-Teknik). IF sections were mounted with Fluoromount G DAPI (Southern Biotech®).

### Immunofluorescence staining

Antigen retrieval through heat-induced epitope retrieval using citrate buffer was performed on deparaffinised sections of SMG and SLG. PG was not included in further analysis due to no observed radiation damage histologically. All sections were blocked with avidin (10 µg/ml) and biotin (2 mg/ml). The primary antibodies listed in Supplementary Table S1 were incubated over night at 4°C. The secondary antibodies used were goat-anti-rat IgBio, goat-anti-rat IgG AF555, goat-anti-rabbit IgG AF488, goat-anti-rabbit IgG AF555, and donkey-anti-guinea pig IgG Cy2 (Supplementary Table S1). Cell nuclei were stained with DAPI (Southern Biotech®).

### Histological analyses

Images were acquired on a Nikon eclipse Ti2 microscope with a CSU-W1 SoRa spinning disk (Yokogawa) with a Prism BSI camera (Photometrics®) using 40x air immersion objective (NA 0.95), 60x water immersion objective (NA 1.33) or 100x oil immersion objective (NA 1.52). A subset of samples were used for K19/K5/Nkcc1/AQP5/MIST1/Ki67 (*n* = minimum 5 for control and irradiated groups) and αSMA/Vimentin/CD45 (*n* = minimum 3 for control and irradiated groups) owing to limited tissue availability. NIS Elements imaging software (Version 6.10.02) was used to analyse the images using the built-in General Analysis 3.

### Statistical analyses

Statistical analyses were performed in Prism 10 for Windows (Version 10.1.2, GraphPad Software, LLC). Shapiro Wilkinson normality test was used to test normal distribution. If all groups passed the normality test, ordinary one-way analysis of variance (ANOVA) and Tukey’s multiple comparisons test was used to analyse differences between groups. If one or more group failed the normality test, Kruskal–Wallis test with Dunn’s multiple comparisons test was used to determine differences between groups. A significance level of 0.05 was used for all comparisons.

## Results

**Figure 1:**
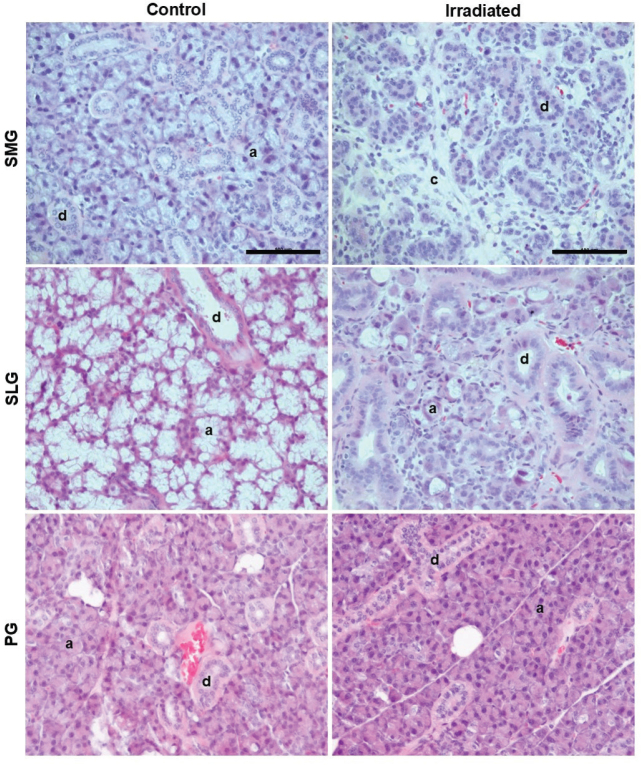
Acinar cell atrophy in SMG and SLG, but not PG of irradiated mice. Representative images of hematoxylin and eosin (HE) stained sections 100 days after fractionated irradiation in controls and irradiated (IR) mice. HE sections of IR and control submandibular (SMG), sublingual (SLG) and parotid (PG) glands, shows atrophy of acinar cells and their replacement by connective tissue in irradiated SMG and SLG but not PG. Scale bar is 100 µm. a = acinar cells, d = ducts, c = connective tissue.

**Figure 2:**
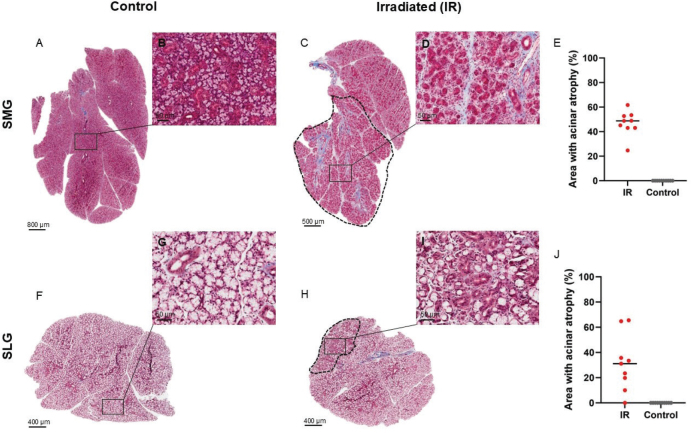
Acinar atrophy was only located in one region of each irradiated (IR) gland. A, C, F, H: Representative overview images of the whole left gland of the submandibular (SMG) and the sublingual (SLG) glands stained with Masson trichrome staining that stains connective tissue blue, which there is more of around the excretory ducts. The black dotted line (in C and H) shows the area with atrophic acinar cells in SLG or complete loss of acinar cells in SMG. B, D, G, I: Zoomed-in part of control and IR SMG and SLG. Scale bar is 50 µm. E, J: Percentage atrophic area of control and IR SMG and SLG, where each red and grey dot represents an animal.

HE stained sections of the major salivary glands showed areas with complete loss of acinar cells in irradiated SMG and areas with acinar cells of reduced sizes in irradiated SLG. In contrast, such changes were not seen in irradiated PG nor in the controls (Figure 1). Fewer and smaller mucous acinar cells were also demonstrated in irradiated versus control mice through AB staining (Supplementary Figure S2). Interestingly, atrophy of acinar cells was observed only in one single area of each irradiated gland and constituted 25–62% of the SMG and 0–66% of the SLG (Figure 2). In all irradiated SMG, the atrophic region was located in the caudal part of the gland, surrounding excretory ducts.

**Figure 3:**
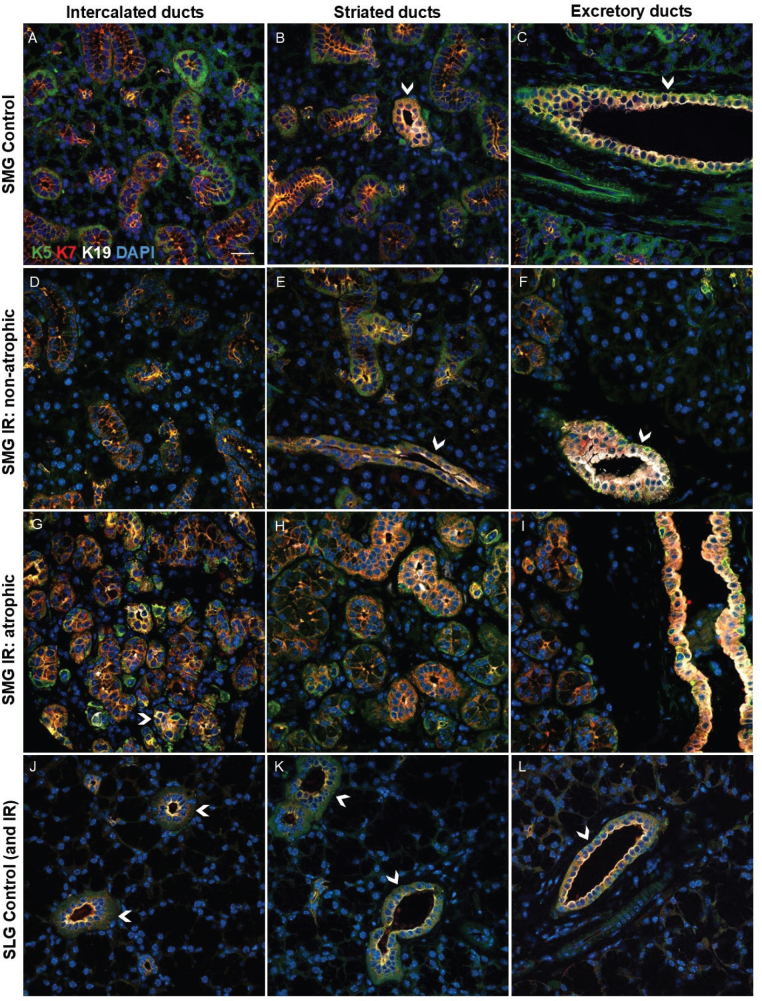
Keratin expression in the intercalated, striated, and excretory ducts of SMG and SLG. Keratin 5 (K5) is shown in green, keratin 7 (K7) in red, keratin 19 (K19) in white, and all nuclei in blue (DAPI). A-F: In control and non-atrophic irradiated (IR) submandibular gland (SMG) all ducts were labelled with K5 and K7, while only the striated and excretory ducts were also labelled with K19. Arrowheads in B, C, E, and F show examples of striated and excretory ducts that are green, red, and white and thereby express all the three keratins. G-I: In IR atrophic SMG some intercalated ducts were also seen with K19 expression in contrast to non-atrophic regions and controls where all intercalated ducts only expressed K5 and K7. Arrowhead in G shows an example of an intercalated duct that is green, red, and white and thereby express all the three keratins. J-L: In control and IR SLG (IR not shown as it appeared similar to control SLG, and we were not able to differentiate between atrophic and non-atrophic regions of IR SLG in this subset of analysis) all ducts are labelled with K5, K7, and K19 (arrowheads). Scale bar is 20 µm.

**Figure 4:**
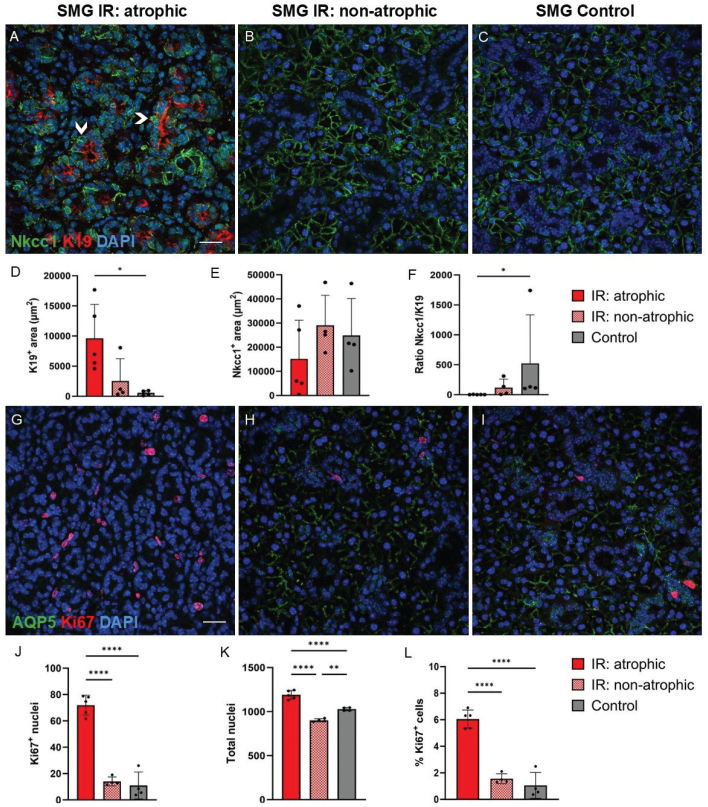
Acinar, ductal, and proliferating cells in atrophic and non-atrophic regions of irradiated (IR) and control SMG. Acinar cells are labelled green with AQP5 or Nkcc1, duct cells or proliferating cells are labelled red with K19 or Ki67, and all nuclei are blue (DAPI). (A–F): Increased expression of K19 was found in the atrophic regions of IR glands compared to control glands. Some K19^+^ cells also co-expressed Nkcc1. Arrowheads in A show examples of duct-like cells that express green Nkcc1 on the basolateral membrane and red K19 on the apical membrane. (G–L): Increased number and percentage of Ki67^+^ cells (relative to all DAPI^+^ cells) were found in the atrophic regions of IR SMG compared to non-atrophic regions in IR SMG and control SMG. Scale bar is 20 µm. Data are presented as mean ± SD. Asterisks are shown between groups that demonstrate statistically significant differences. *p ≤ 0.05, **p ≤ 0.01, ****p ≤ 0.0001. Each black dot represents an animal.

To investigate the ductal cells of the salivary glands, we performed immunofluorescent staining using several ductal cell markers, specifically keratin 5 (K5), keratin 7 (K7), and keratin 19 (K19). These three keratins have been used in similar studies, which can make comparisons easier. They have also been observed to be involved in cellular plasticity of the salivary glands previously and were therefore of particular interest [7, 11, 17]. We also utilised various acinar cell markers including sodium potassium chloride cotransporter 1 (Nkcc1), aquaporin 5 (AQP5), and muscle, intestine, and stomach expression 1 (MIST1), to gain a comprehensive understanding of the acinar cells. Nkcc1 mediates sodium-driven chloride influx into acinar cells [18, 19], while AQP5 is a membrane water channel protein that mainly permeate water [20]. MIST1 is expressed in acinar cells of pancreatic and salivary glands and is important in maintaining the secretory phenotype [21–23]. All these acinar cell markers are also commonly used in similar studies [4, 5, 7–11]. In controls and non-atrophic irradiated SMG, all ducts expressed K5 and K7, while K19 was expressed by the striated and excretory ducts only, but not by the intercalated ducts (Figure 3A–F). In atrophic regions of irradiated SMG, area exhibiting K19 expression expanded, indicating that a greater number of cells were expressing K19. Here, even some K19^+^ intercalated ducts were observed (Figure 3G–I). In comparison, all ducts including intercalated ducts, in control and irradiated SLG were positive for K5, K7, and K19 (Figure 3J–L). In atrophic regions of irradiated SMG, some duct-like cells showed K19 expression on the apical membrane, and Nkcc1 expression on the basolateral membrane (Figure 4A). This was not seen in non-atrophic regions and controls (Figure 4B–D). We did not observe a statistically significant decrease of Nkcc1 expression in the atrophic regions (Figure 4E), to account for the acinar atrophy that was seen by HE, AB, and MT staining. However, the ratio of increased K19 expression versus the trend to decreased Nkcc1 expression showed statistically significant Nkcc1/K19 ratio in the atrophic regions of irradiated SMG compared to control SMG (Figure 4F).

In the atrophic regions of irradiated SMG, we found a higher number of proliferating cells (Ki67^+^) compared to non-atrophic regions and controls (Figure 4G–J). To account for the difference in cell density between the groups (Figure 4K), the percentage of Ki67^+^ proliferating cells relative to the total number of cells (DAPI^+^ cells) was measured and was also found to be increased in atrophic regions compared to non-atrophic regions and controls (Figure 4L).

**Figure 5:**
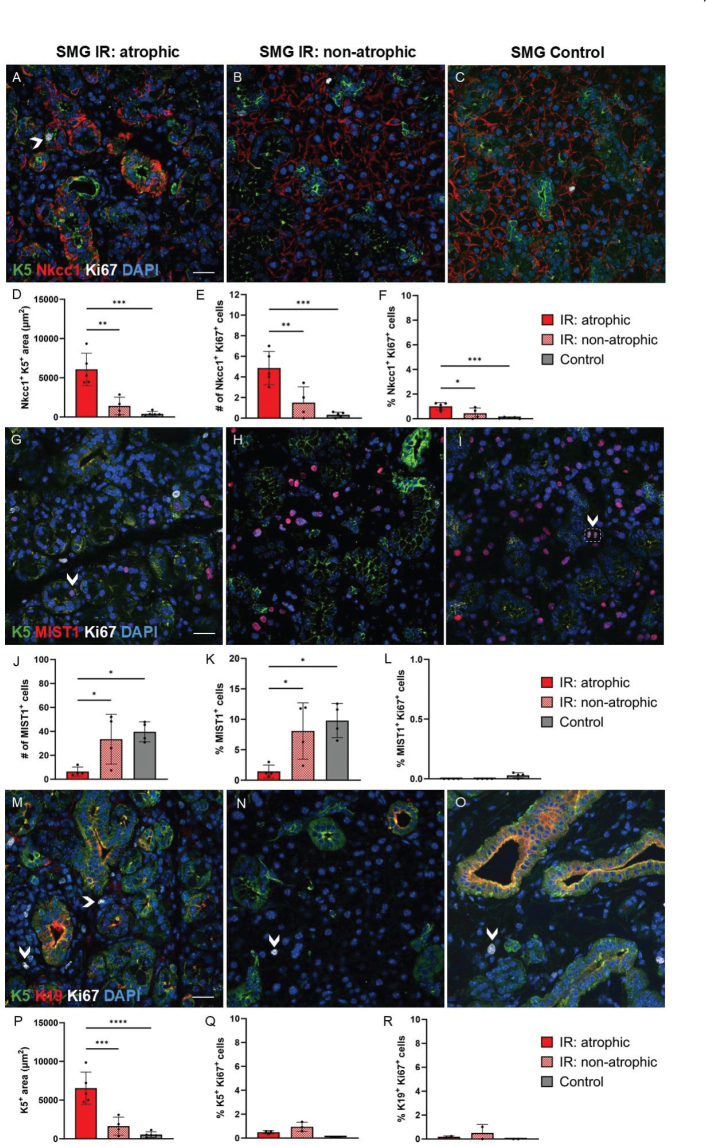
Ki67 expression in acinar and ductal cells of atrophic and non-atrophic regions of irradiated (IR) and control submandibular glands (SMG). Acinar cell markers (Nkcc1 and MIST1) or the ductal cell marker Keratin 19 (K19) are shown in red, while the more general duct cell marker Keratin 5 (K5) is shown in green, proliferating cells are shown in white (Ki67), and all nuclei are shown in blue (DAPI). (A–C): In atrophic regions of IR SMG some K5^+^ duct-like cells also expressed Nkcc1 and Ki67, while this was not seen in non-atrophic regions or controls. Arrowhead in A shows an example of a K5^+^Nkcc1^+^Ki67^+^ cell, where green K5 is expressed on the apical membrane, red Nkcc1 on the basolateral membrane, and white Ki67 in the nucleus. (D): More Nkcc1^+^K5^+^ cells were seen in atrophic regions of IR SMG than non-atrophic regions and control SMG. (E–F): Increased number and percentage of proliferating Nkcc1^+^ acinar cells (Nkcc1^+^Ki67^+^ cells relative to all DAPI^+^ cells) were found in atrophic regions of IR SMG compared to non-atrophic regions and control SMG. (G–L): Reduced number of MIST^+^ acinar cells were observed in atrophic regions of IR SMG compared to non-atrophic regions and control SMG, but a few MIST1^+^ acinar cells co-expressed the ductal cell marker K5. Arrowhead in G shows a K5^+^MIST1^+^ cell, where green K5 is expressed on the membrane while red MIST1 is expressed in the nucleus. Very few MIST1^+^Ki67^+^ cells were seen in all groups (an example shown with arrowhead and dotted white box in I, where red MIST1 and white Ki67 are both expressed in the nuclei of two daugther cells from a newly divided cell). (M–R): Increased K5^+^ area was found in atrophic regions of IR SMG compared to non-atrophic regions and control SMG, however K5^+^ or K19^+^ cells showed the same proliferation rate in all groups. Arrowheads in M, N, and O show examples of proliferating cells (white Ki67^+^) that are not ductal cells (K5^+^ or K19^+^). Scale bar is 20 µm. Data are presented as mean ± SD. Asterisks are shown between groups that demonstrate statistically significant differences. *p ≤ 0.05, **p ≤ 0.01, ***p ≤ 0.001, ****p ≤ 0.0001. Each black dot represents an animal.

**Figure 6:**
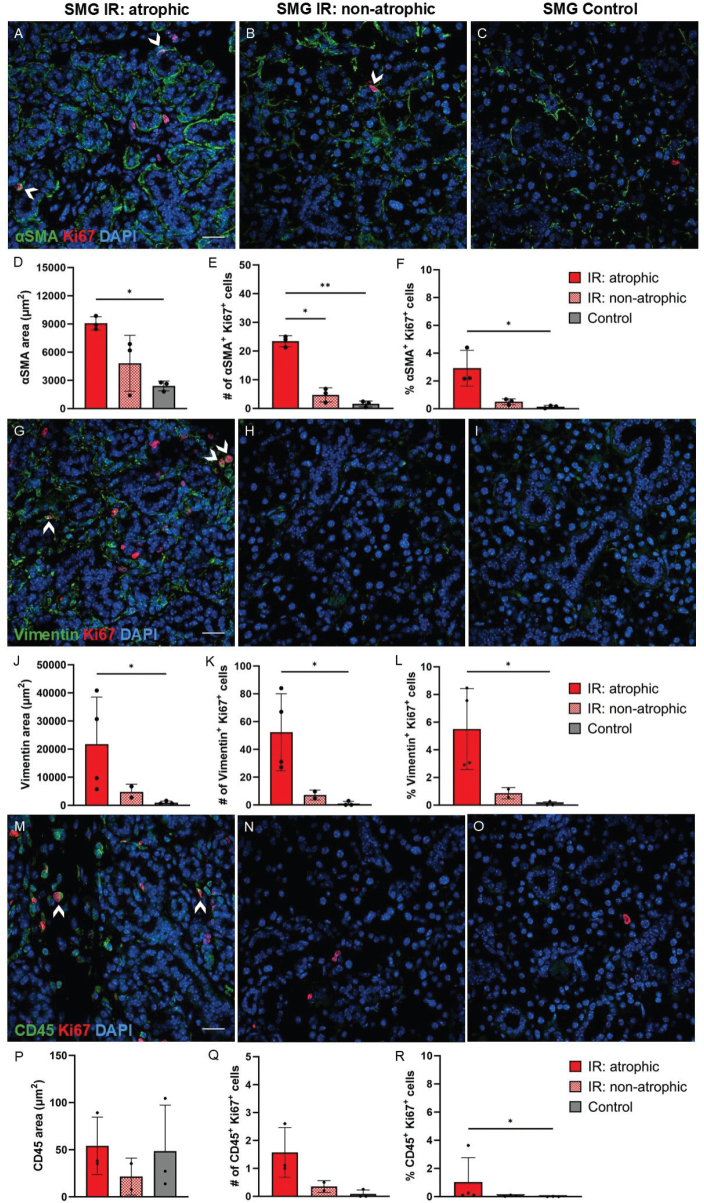
Ki67 expression in myoepithelial cells, fibroblasts, and leukocytes of atrophic and non-atrophic regions of irradiated (IR) and control SMG. Myoepithelial cells (αSMA), fibroblasts (Vimentin) and leukocytes (CD45) are shown in green, proliferating cells are shown in red (Ki67), and all nuclei are shown in blue (DAPI). (A–F): Increased αSMA area, number and percentage of αSMA^+^ Ki67^+^cells (relative to all DAPI^+^ cells) were seen in atrophic regions of IR SMG compared to control SMG. Arrowheads in A and B show examples of proliferating myoepithelial cells, where green αSMA is expressed in the cytoplasm while red Ki67 is expressed in the nucleus. (G–L): Increased vimentin area, number and percentage of vimentin^+^ Ki67^+^cells (relative to all DAPI^+^ cells) were seen in atrophic regions of IR SMG compared to control SMG. Arrowheads in G show examples of proliferating fibroblasts, where green vimentin is expressed in the cytoplasm while red Ki67 is expressed in the nucleus. (M–R): Increased percentage of CD45^+^ Ki67^+^cells (relative to all DAPI^+^ cells) were seen in atrophic regions of IR SMG compared to control SMG. Arrowheads in M show examples of proliferating leukocytes, where CD45 is expressed in the cytoplasm while red Ki67 is expressed in the nucleus. Scale bar is 20 µm. Data are presented as mean ± SD. Asterisks are shown between groups that demonstrate statistically significant differences. *p ≤ 0.05, **p ≤ 0.01. Each black dot represents an animal.

We performed further immunofluorescent staining to investigate whether the proliferating cells in the atrophic regions of irradiated SMG were acinar and/or ductal cells using the previously mentioned acinar and ductal cell markers together with the proliferation marker, Ki67. We found increased percentage of proliferating acinar cells (Nkcc1^+^ Ki67^+^ cells relative to all DAPI^+^ cells) in the atrophic regions of irradiated SMG compared to non-atrophic regions and control SMG (Figure 5A–F). Moreover, reduced number of MIST1^+^ acinar cells was seen in the atrophic regions compared to non-atrophic regions and controls. Very few proliferating MIST1^+^ acinar cells (MIST1^+^Ki67^+^) were seen in all groups (Figure 5G–L). In addition, the increased areas with K5 and K19 expression in the atrophic regions did not translate to more proliferating K5^+^ or K19^+^ duct cells, as they showed similar proliferation rate as in non-atrophic regions and controls (Figure 5M–R). We also wanted to elucidate whether the proliferating cells where other cell types than acinar and ductal cells and therefore stained with markers for myoepithelial cells (αSMA), fibroblasts (vimentin), and leukocytes (CD45). Increased areas with αSMA and vimentin expression observed in atrophic regions of irradiated SMG compared to control SMG translated to higher percentages of proliferating myoepithelial cells (αSMA^+^ Ki67^+^ cells relative to all DAPI^+^ cells) and fibroblasts (vimentin^+^ Ki67^+^ cells relative to all DAPI^+^ cells) (Figure 6A–L). We also found a higher percentage of proliferating leukocytes (CD45^+^ Ki67^+^ cells relative to all DAPI^+^ cells) in the atrophic regions of irradiated SMG compared to control SMG (Figure 6M–R). Taken together, this shows that although most of the proliferating cells in the atrophic regions of irradiated SMG were myoepithelial cells, fibroblasts or leukocytes, some were also acinar cells (Nkcc1^+^Ki67^+^).

**Figure 7:**
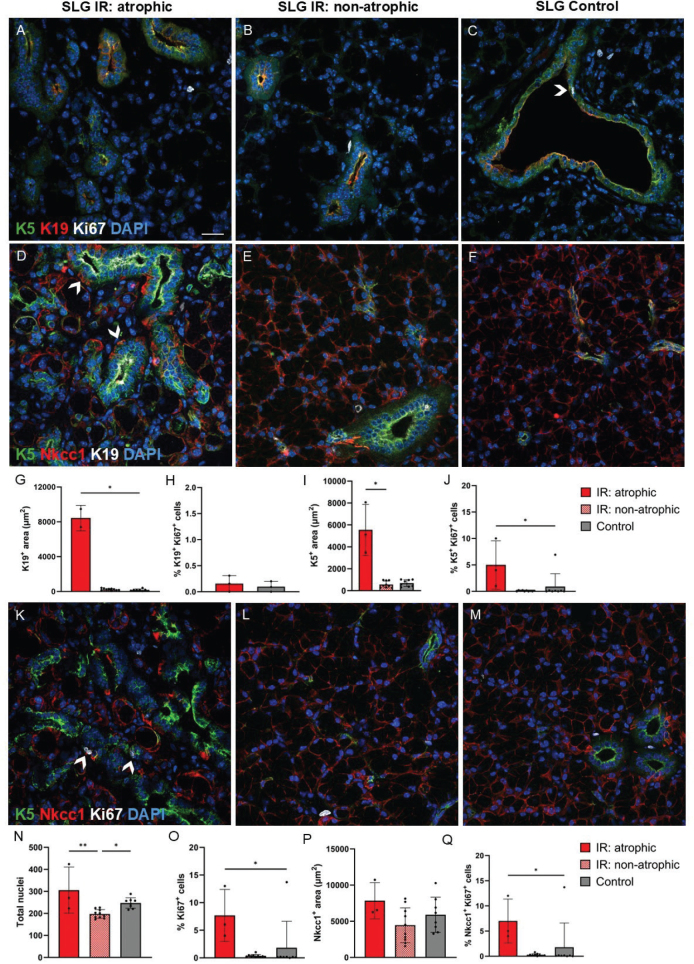
Ki67 expression in acinar and ductal cells in atrophic and non-atrophic regions of irradiated (IR) and control sublingual glands (SLG). **(**A–C): The ductal cell marker Keratin 19 (K19) is shown in red, while the more general duct cell marker Keratin 5 (K5) is shown in green, proliferating cells are shown in white (Ki67), and all nuclei are shown in blue (DAPI). The arrowhead in C shows an example of a green and red K5^+^ K19^+^ ductal cell that co-expresses white Ki67 in the nucleus. (D–Q): The acinar cell marker, Nkcc1 is shown in red, K5 is shown in green, K19 or Ki67 is shown in white, and all nuclei are shown in blue (DAPI). We found increased K19 and K5 expression in atrophic regions of IR SLG compared to control SLG. Some of the K19^+^ and/or K5^+^ duct-like cells also co-expressed Nkcc1. Arrowheads in D show duct-like cells that express green K5 and white K19 on the apical membrane and red Nkcc1 on the basolateral membrane. Increased percentage of proliferating cells (Ki67^+^ cells relative to all DAPI^+^ cells) were also found in the atrophic regions of IR SLG compared to control SLG. Some of these Ki67^+^ cells were proliferating ductal (K5^+^ Ki67^+^) and/or acinar cells (Nkcc1^+^ Ki67^+^). The arrowheads in K show examples of proliferating cells (white Ki67^+^) that express both green K5 and red Nkcc1. Scale bar is 20 µm. Data are presented as mean ± SD. Asterisks are shown between groups that demonstrate statistically significant differences. *p ≤ 0.05, **p ≤ 0.01. Each black dot represents an animal.

Similar to what was found in SMG, we also observed increased areas with K5 and K19 expression in atrophic regions of irradiated SLG compared to control SLG (Figure 7A–I). Some of these K5^+^ and/or K19^+^ cells also expressed Nkcc1 and Ki67. Furthermore, increased percentage of proliferating cells (Ki67^+^ cells relative to all DAPI^+^ cells) was found in atrophic regions of SLG compared to controls. In contrast to SMG, the atrophic regions in SLG were smaller and not possible to visualise in all IF sections in SLG due to larger inter-individual variations and smaller glands than SMG. Therefore, there is more uncertainty regarding which cells are proliferating in the atrophic regions of SLG than SMG. We found a higher percentage of proliferating acinar cells (Nkcc1^+^ Ki67^+^ cells relative to all DAPI^+^ cells) and ductal cells (K5^+^ Ki67^+^ cells relative to all DAPI^+^ cells) in the atrophic SLG compared to control SLG (Figure 7J–Q). Regarding Ki67 co-expression with αSMA, vimentin, and CD45, however, we were unable to differentiate between non-atrophic and atrophic regions of irradiated SLG in that subset of analysis (Figure S3) and cannot conclude whether the proliferating cells in atrophic regions of irradiated SLG were myoepithelial cells, fibroblasts, or leukocytes.

## Discussion and conclusion

We have previously shown that the current fractionated irradiation set-up and mouse model resulted in increased salivary gland fibrosis and reduced saliva production [12, 13]. In this study using the exact same fractionation scheme and dose as previously, we found atrophy of acinar cells in irradiated SMG and SLG, but not in PG. Interestingly, atrophy of acinar cells was observed only in one single area of each irradiated gland. These atrophic areas showed distinct cellular and molecular compositions compared to non-atrophic areas of the same irradiated glands and control glands. Notably, increased expression of the ductal cell markers K19 and K5, along with increased percentages of proliferating myoepithelial cells, fibroblasts, leukocytes, and acinar cells (Nkcc1^+^) were observed in the atrophic regions compared to non-atrophic regions and control glands. In addition, some of the K19^+^ and K5^+^ duct-like cells also co-expressed the acinar cell marker Nkcc1. This implies a cellular plasticity where ductal cells adopt an acinar cell phenotype upon radiation injury potentially to aid in regenerating the damaged acinar cell population.

This study demonstrated loss of acinar cells in SMG and SLG, but no loss of acinar cells in the PG, at day 100 after irradiation. A study using Wistar rats showed larger effects in the SMG than in the PG 240 days after fractionated irradiation [24]. In that work it was shown that after single dose irradiation, SMG and PG responded rather similarly, but their differences became evident after fractionated irradiation, especially at late time points after radiation. The investigators observed more loss of acinar cells in SMG than in PG, which is consistent with what we observed. Taken together, this may indicate that PG is more radioresistant to fractionated irradiation compared to SMG and SLG, at least at late time points in mice and rats. However, as we avoided irradiation of the eyes and brain, the cranial part of the PG therefore might not have been in the radiation field, due to its anatomical location. Since we did not observe radiation-induced damage in PG histologically, we investigated the damage observed in SMG and SLG further using immunofluorescent staining.

Acinar atrophy was only observed in one single area of each irradiated gland of SMG and SLG. In the irradiated SMG the atrophic area was consistently located in the caudal part of the gland seemingly closely situated to the excretory ducts. Increased radiosensitivity has been found in humans and rats near the major excretory ducts, which was believed to be caused by c-KIT^+^ stem cells residing in these ducts [25, 26]. However, more recent long-term lineage tracing studies have shown that homeostasis of the adult salivary gland does not rely on multipotent stem cells [4, 5, 27]. Additionally, c-KIT may not be a reliable marker for salivary gland stem cells [28]. In other words, the previous assumption that salivary gland stem cells resided in the major ducts is debated [6] and might not be the only reason why we see acinar atrophy only in the caudal part of SMG. As the radiation field included the entire SMG and SLG [14], the regional atrophy cannot be caused by a heterogeneous dose distribution either. Therefore, the most likely explanation for the regional atrophy in the glands is a difference in radiosensitivity. Whether the more radiosensitive area is due to a larger proportion of progenitor cells being located there is, however, not completely known due to the recent evolvement in the understanding of salivary gland stem/progenitor cells [1, 6].

In a recent study K19 was shown in excretory, striated, and intercalated ductal cells in SMG of normal male C57BL/6 mice, but with weak staining in the intercalated ducts [17]. In the present study we did not observe K19 expression in the intercalated ducts of SMG in normal female C57BL/6JRj mice. There might be several reasons for this inconsistency, such as different K19 antibodies used (both monoclonal antibodies, but different clones), that male and female mice show hormone-driven sexual dimorphism in the SMG [1, 29], and that there might be possible differences between C57BL/6 substrains [30]. Documentation of K5, K7, and K19 expressions in the different ductal cell compartments in normal murine SMG and SLG is lacking, and this study is therefore an important evaluation of both normal and irradiated glands.

In contrast with the long-standing assumption that a stem cell population supports adult salivary gland homeostasis, emerging evidence indicate that the acinar and ductal cell populations are maintained by lineage-restricted progenitor cells, where the different cell compartments replenish themselves [1, 4–6]. After stress or injury, however, there has been observed cellular plasticity between the different cell compartments in the salivary glands, where ductal cells adopt an acinar cell phenotype possibly to aid in regeneration of the damaged acinar cell population. This has been shown after duct ligation in mice [7, 8], irradiation in mice [5, 9, 10], and after radiotherapy in humans [11]. Duct ligation is a method often used to study atrophy and regeneration of salivary glands and has several similarities with irradiation models [1]. Duct ligation results in a reversible loss of acinar cells, while radiation injury causes a permanent loss of most acinar cells [9, 31]. In previous studies showing evidence of cellular plasticity, increased expression of K7 and K19 have been observed in SMG of male C57BL/6 mice after duct ligation [7], and increased K5 expression has been observed in human SMG and PG after radiotherapy [11]. In these K7^+^, K19^+^, or K5^+^ duct-like cells some of them also co-expressed acinar cell markers (MIST1 and/or AQP5 and/or Nkcc1). In our study, we observed increased K5 and K19 expression, where several of these K5^+^ and/or K19^+^ duct-like cells also co-expressed the acinar cell marker, Nkcc1. Our data are therefore in line with the previously mentioned studies [7, 11] and support the hypothesis regarding cellular plasticity in salivary glands after radiation injury.

In atrophic regions of irradiated SMG and SLG we found an increased percentage of proliferating cells (Ki67^+^) compared to non-atrophic regions and controls. Most of these proliferating cells were myoepithelial cells, fibroblasts, or leukocytes, but interestingly, some of them were also acinar cells (Nkcc1^+^). In another study, Luitje and coworkers found an increased number of proliferating cells (Ki67^+^) in human SMG and PG at late time points after radiotherapy [11]. Similar to what was observed in this study, they also found that some of these proliferating cells were acinar cells (Nkcc1^+^). Moreover, they saw an increase of proliferative acinar cells after radiotherapy compared to controls, indicating that acinar cells remain their proliferative capacity in human salivary glands after radiotherapy, which is in line with our observations. Notably, we also observed that some of the K5^+^ Nkcc1^+^ duct-like cells co-expressed Ki67. Despite the loss of acinar cells we found in the irradiated glands, some of the cells expressing both ductal and acinar cell markers show proliferative activity 100 days after irradiation. This indicates a regenerative capacity due to cellular plasticity in salivary glands after severe radiation injury.

To conclude, this work is a thorough histological assessment of the cellular and molecular changes in the SMG and SLG of irradiated mice. In the atrophic regions of irradiated glands, we observed signs of cellular plasticity where duct-like cells expressed both ductal cell markers and an acinar cell marker. This indicates that ductal cells adopt an acinar cell phenotype upon radiation-induced damage possibly to aid in regeneration of the lost acinar cells. In addition, we found proliferating cells co-expressing an acinar cell marker, which suggests some degree of regenerative potential of salivary gland cells after irradiation. Even though the observed cellular plasticity and potential regeneration might not result in full restoration of the salivary gland function in this study due to extensive fibrosis and loss of acinar cells [12, 13], increased knowledge on this subject can lead to an understanding of how to exploit plasticity for the repair of damaged salivary glands following radiotherapy.

## Supplementary Material



## Data Availability

The data that support the findings of this study are available from the corresponding author, ISJ, upon reasonable request.
