# Supplementary material for: Regional atrophy, cellular plasticity, and regenerative potential in irradiated murine salivary glands
Source: Acta Oncol. 2025 Jul 23;64:44012. doi: 10.2340/1651-226X.2025.44012 (PMC12305691; doi:10.2340/1651-226X.2025.44012)
Supplement: Supplementary file 1 [file AO-64-44012-s1.pdf]

Supplementary material has been published as submitted. It has not been copyedited, or typeset by Acta Oncologica

Supplementary materials

Staining of tissues

Hematoxylin and eosin staining
Progressive hematoxylin and eosin (HE) staining was used by immersing the slides in Mayer's hematoxylin for 1 minute, followed by 2 minutes in 0.25 % hexamine and finally by 3 minutes in eosin. Between every staining step, the slides were washed in running tap water for 5 minutes.

Alcian blue staining
Alcian blue staining (AB) was used to detect mucous acinar cells as it stains acidic mucins blue. The slides were incubated with 3% acetic acid for 10 minutes at room temperature, washed in distilled water and incubated with Alcian blue (8GX, Sigma-Aldrich) for 30 minutes. Furthermore, the slides were washed in tap water and counterstained with nuclear fast red for 1 minute.

Masson trichrome staining
Masson trichrome (MT) staining was used to detect fibrosis as it stains fibrous tissue blue. MT staining was performed using a ready-to-use kit (Trichrome Stain kit, Abcam) on a central section of each SMG and SLG. Briefly, the slides were immersed in preheated Bouin's fluid for 60 minutes at 60 °C. Thereafter, the slides were cooled, washed in tap water for 5 minutes, rinsed in distilled water and stained with Weigert's hematoxylin for 5 minutes. Subsequently, the slides were washed in tap water for 2 minutes and stained with Biebrich scarlet-acid fuchsin for 15 minutes. The slides were then rinsed in distilled water, incubated with phosphomolybdic-phosphotungstic acid for 15 minutes, dyed with aniline blue for 10 minutes, rinsed in distilled water and fixed with 1 % acetic acid for 5 minutes

Table S1: Overview of the primary and secondary antibodies used for immunofluorescence staining

Antibody	Host	Clone	Vendor	Conc.	RRID	Proper citation
αSMA	Rabbit mAb	SP171	Abcam	N/A 1/2000	AB_3675461	Abcam Cat# ab150301, RRID:AB_3675461
AQP5	Rabbit pAb	N/A	Alomone labs	1 mg/ml	AB_2039736	Alomone Labs Cat# AQP-005 RRID: AB_2039736
CD45	Rabbit pAb	N/A	Abcam	1 µg/ml	AB_442810	Abcam Cat# ab10558, RRID:AB_442810
c-KIT (CD117)	Rabbit pAb	N/A	AbboMax	5 µg/ml	AB_3694084	AbboMax Cat# 600-970, RRID: AB_3694084
Keratin 5	Guinea pig pAb	N/A	ProGen	N/A 1/200	AB_2920679	Progen Cat# GP-CK5, RRID:AB_2920679
Keratin 7	Rabbit mAb	CPTC-KRT7-2	DSHB*	0.1 µg/ml	AB_2820265	DSHB Cat# CPTC-KRT7-2, RRID:AB_2820265
Keratin 19	Rat mAb	TROMA-III	DSHB**	0.5 µg/ml	AB_2133570	DSHB Cat# TROMA-III, RRID:AB_2133570
Ki67	Rabbit mAb	SP6	Invitrogen	0.15 µg/ml	AB_10979488	Thermo Fisher Scientific Cat# MA5-14520, RRID:AB_10979488
Ki67	Rat mAb	SolA15	eBioscience	0.5 µg/ml	AB_10854564	Thermo Fisher Scientific Cat# 14-5698-82, RRID:AB_10854564
MIST1	Rabbit pAb	N/A	Atlas Antibodies	0.16 µg/ml	AB_2680172	Atlas Antibodies Cat# HPA047834, RRID:AB_2680172
Nkcc1	Rabbit pAb	N/A	Invitrogen	1 µg/ml	AB_2806950	Thermo Fisher Scientific Cat# PA5-95145, RRID:AB_2806950
Vimentin	Rabbit mAb	EPR3776	Epitomics	N/A 1/500	AB_2216258	Abcam Cat# 2707-1, RRID:AB_2216258
Gt-a-Rat IgBio	Goat pAb	N/A	Southern Biotechnology	2.5 µg/ml	AB_2795803	SouthernBiotech Cat# 3010-08, RRID:AB_2795803
Gt-a-Rat IgG AF555	Goat pAb	N/A	Invitrogen	5 µg/ml	AB_2535855	Thermo Fisher Scientific Cat# A-21434, RRID:AB_2535855
Gt-a-Rb IgG AF488	Goat pAb	N/A	Invitrogen	5 µg/ml	AB_2536097	Thermo Fisher Scientific Cat# A-27034, RRID:AB_2536097
Gt-a-Rb IgG AF555	Goat pAb	N/A	Invitrogen	5 µg/ml	AB_2535849	Thermo Fisher Scientific Cat# A-21428, RRID:AB_2535849
Dk-a-GP IgG Cy2	Guinea Pig pAb	N/A	Jackson Immuno-Research Labs	7.5 µg/ml	AB_2340467	Jackson ImmunoResearch Labs Cat# 706-225-148, RRID:AB_2340467

* CPTC-KRT7-2 was deposited to the DSHB by Clinical Proteomics Technologies for Cancer (DSHB Hybridoma Product CPTC-KRT7-2)
** TROMA-III was deposited to the DSHB by Kemler, R. (DSHB Hybridoma Product TROMA-III)
Abbreviations: αSMA (α-smooth muscle actin), AQP5 (aquaporin 5), MIST1 (muscle, intestine, and stomach expression 1), Nkcc1 (sodium potassium chloride cotransporter 1), pAb (polyclonal antibody), mAb (monoclonal antibody), Gt-a-Rb (goat-anti-rabbit), Dk-a-GP (donkey-anti-guinea pig).

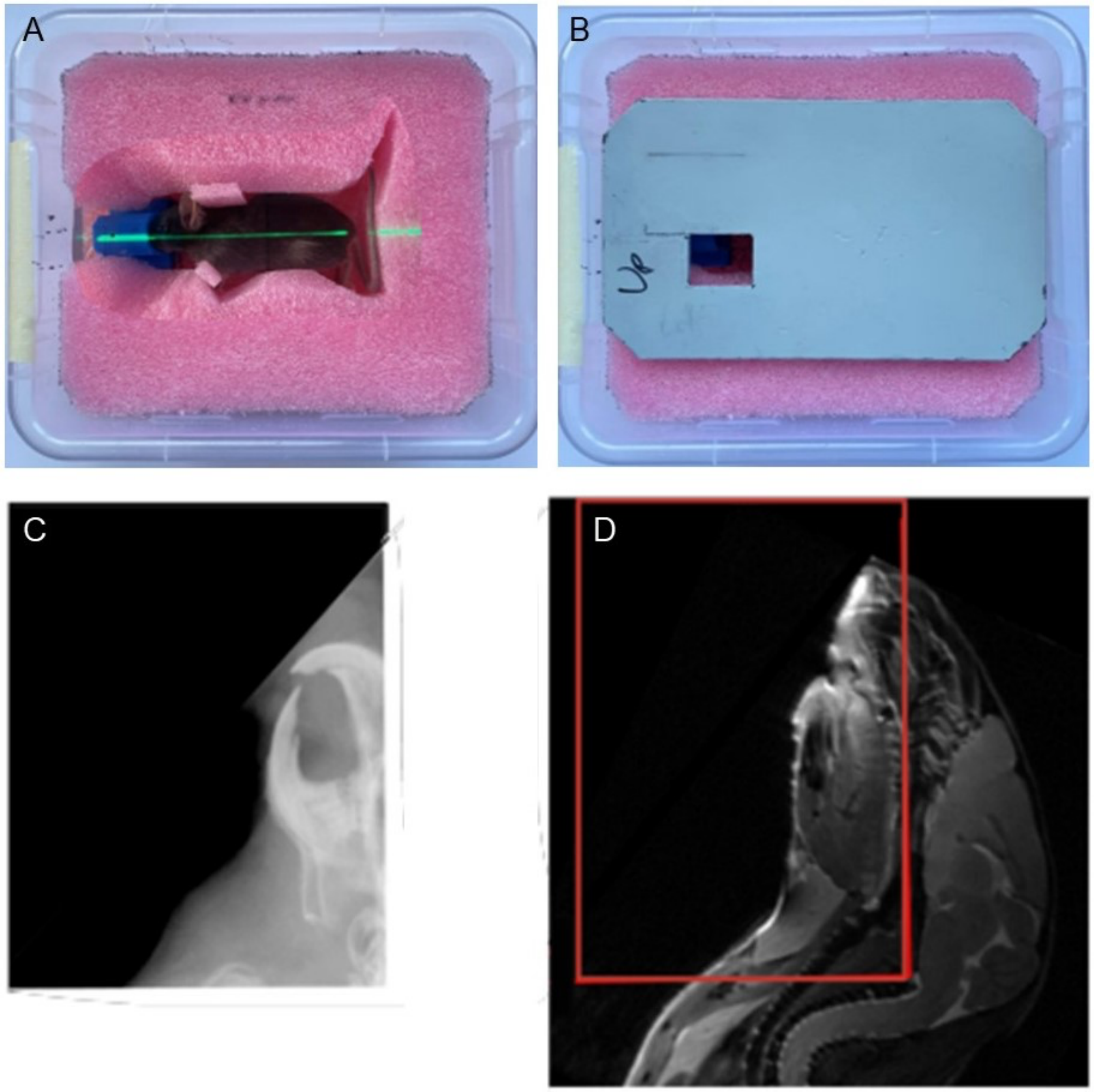

Figure S1. Experimental set-up of the X-ray irradiation procedure
Anaesthetised mouse positioned for irradiation (A) without collimator and (B) with custom-made lead collimator to obtain the correct radiation field. (C) X-ray image of the radiation field depicting the mandible. (D) MR image of the radiation field depicting the oral cavity and major salivary glands (the submandibular gland can be easily seen on the MRI below the tongue inside the oral cavity) inside the radiation field.

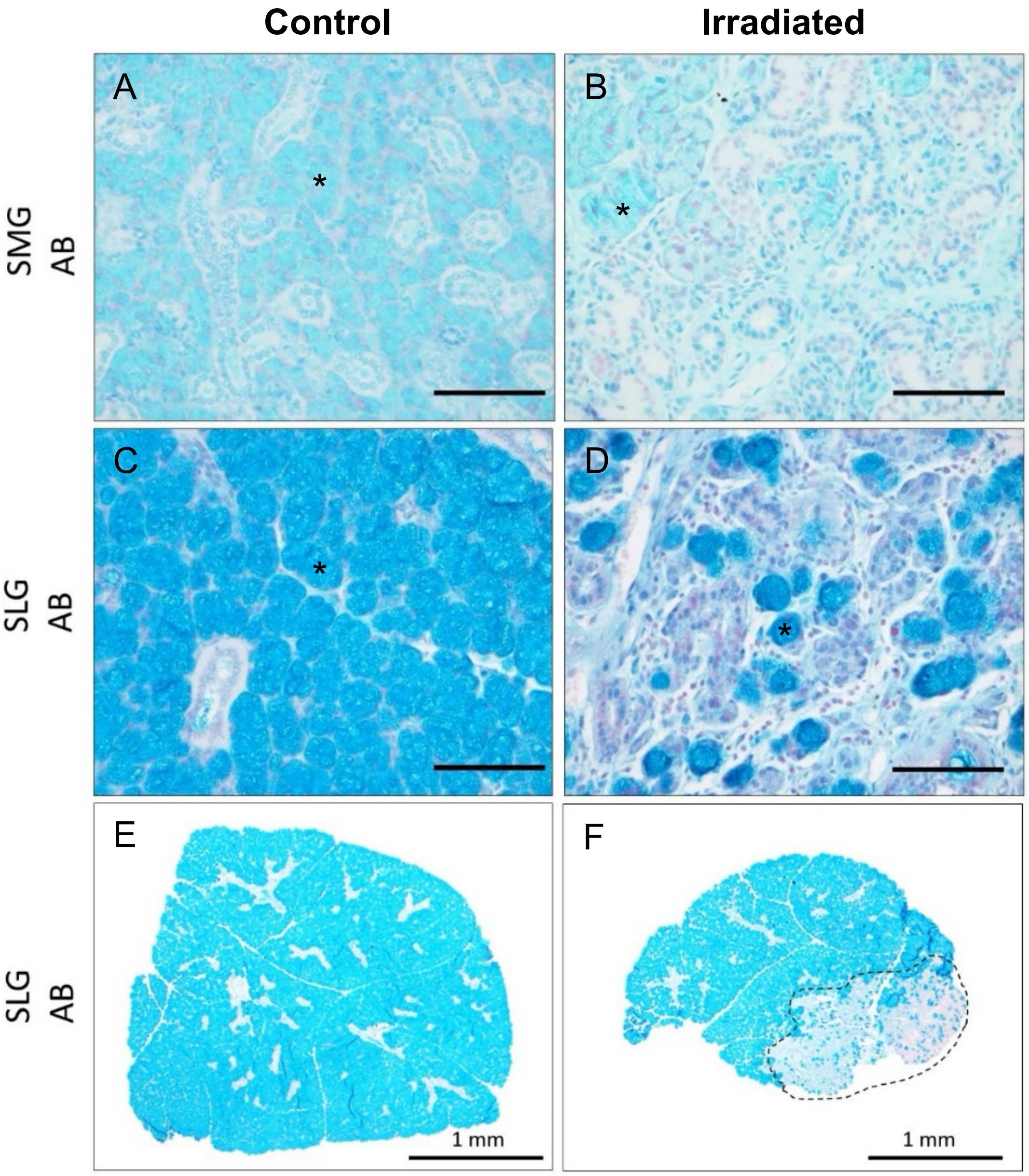

Figure S2. Decreased number of mucous acinar cells in irradiated SMG and SLG
Representative images of alcian blue (AB) stained sections of the submandibular (SMG) and sublingual glands (SLG) in controls and irradiated mice. A-D: Mucous acinar cells are stained blue with alcian blue, while cell nuclei are stained red with nuclear fast red. A reduced number of blue mucous acinar cells are seen in the irradiated sections (asterisks show the mucous acinar cells). Scale bar is 100 µm. E-F: Overview images of SLG (control and irradiated). The focal area (dotted line in F) of the irradiated SLG is stained less blue with AB staining both in comparison to the rest of the SLG tissue and the control.

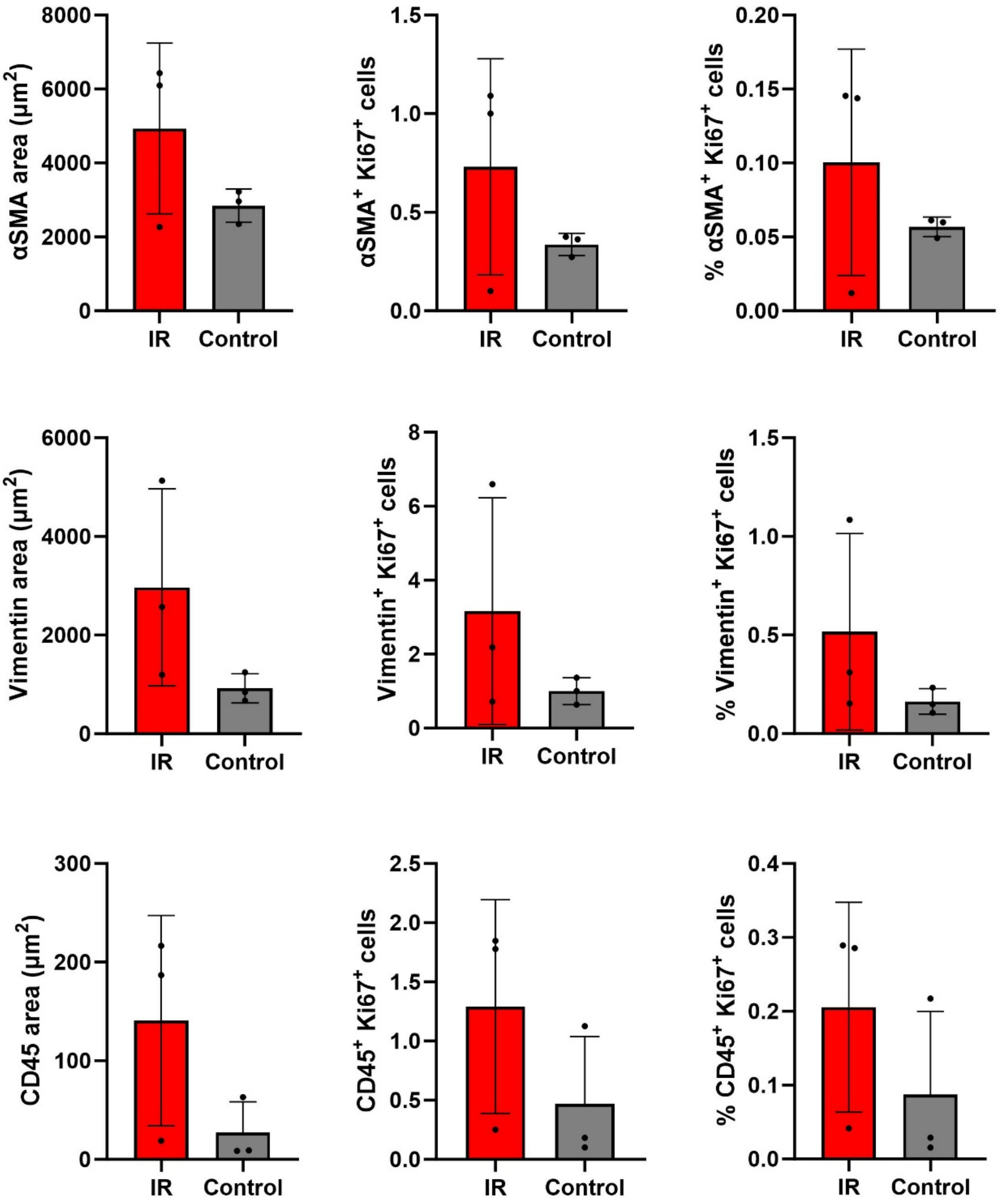

Figure S3. Ki67 expression in myofibroblasts, fibroblasts, and leukocytes in irradiated (IR) and control sublingual glands. Myofibroblasts (αSMA), fibroblasts (Vimentin) and leukocytes (CD45) co-expression with the proliferation marker Ki67 in IR and control SLG. In this subset of analysis, we were unable to differentiate between non-atrophic and atrophic regions of IR SLG.
